# The Proportion Cured of Patients with Resected Stage II–III Cutaneous Melanoma in Sweden

**DOI:** 10.3390/cancers13102456

**Published:** 2021-05-18

**Authors:** Hanna Eriksson, Deborah Utjés, Roger Olofsson Bagge, Peter Gillgren, Karolin Isaksson, Jan Lapins, Inkeri Leonardsson Schultz, Johan Lyth, Therese M.-L. Andersson

**Affiliations:** 1 Department of Oncology and Pathology, Karolinska Institutet, 17177 Stockholm, Sweden; 2 Cancer Theme, Medical Unit Head-Neck-, Lung- and Skin Cancer, Skin Cancer Center, Karolinska University Hospital, 17164 Stockholm, Sweden; 3 Children and Women’s Health Theme, Karolinska University Hospital, 17164 Stockholm, Sweden; deborah.utjes@sll.se; 4 Department of Surgery, Institute of Clinical Sciences, Sahlgrenska Academy, University of Gothenburg, 40530 Gothenburg, Sweden; roger.olofsson@gu.se; 5 Department of Surgery, Sahlgrenska University Hospital, 41345 Gothenburg, Sweden; 6 Wallenberg Centre for Molecular and Translational Medicine, University of Gothenburg, 40530 Gothenburg, Sweden; 7 Department of Clinical Science and Education, Karolinska Institutet, 17177 Stockholm, Sweden; peter.gillgren@sll.se; 8 Department of Surgery, Södersjukhuset, 11883 Stockholm, Sweden; 9 Department of Clinical Sciences, Surgery, Lund University, 22100 Lund, Sweden; karolin.isaksson@med.lu.se; 10 Department of Surgery, Central Hospital Kristianstad, 29133 Kristianstad, Sweden; 11 Department of Dermatology, Karolinska Institutet, 17177 Stockholm, Sweden; jan.lapins@sll.se; 12 Department of Dermatology, Skin Cancer Center, Theme Cancer, Karolinska University Hospital, 17164 Stockholm, Sweden; 13 Division of Reconstructive Plastic Surgery, Department of Molecular Medicine and Surgery, Karolinska Institutet, 17177 Stockholm, Sweden; inkeri.leonardsson-schultz@sll.se; 14 Clinic of Reconstructive Plastic Surgery, Karolinska University Hospital, 17164 Stockholm, Sweden; 15 Department of Health, Medicine and Caring Sciences, Linköping University, 58183 Linköping, Sweden; johan.lyth@liu.se; 16 Department of Medical Epidemiology and Biostatistics, Karolinska Institutet, 17177 Stockholm, Sweden; therese.m-l.andersson@ki.se

**Keywords:** stage II–III melanoma, survival ratios, cure proportions, median survival times, uncured patients

## Abstract

**Simple summary:**

Patients diagnosed with stage II–III cutaneous melanoma (CM) are at high risk of recurrences, but the CM-specific survival ranges from approximately 40–70%. Here, the cure proportions and survival among uncured stage II–III CM patients were estimated. The 1- and 5-year relative survival ratios, cure proportions and the median survival times of uncured stage II–III CM patients in Sweden (*n* = 6466) were calculated based on data from the nationwide population-based Swedish Melanoma Register 2005–2013 with a follow-up through 2018. Proportions cured by surgery are low for sub-groups of stage II–III cutaneous melanoma showing that cure analyses can serve as a complement to established survival analyses.

**Abstract:**

Background: Cure proportion represents the proportion of patients who experience the same mortality rate as the general population and can be estimated together with the survival of the proportion experiencing excess mortality (the uncured). The aim was to estimate the cure proportions and survival among uncured stage II–III cutaneous melanoma (CM) patients. Methods: 1- and 5-year relative survival ratios, cure proportions and the median survival times of uncured stage II–III CM patients in Sweden (*n* = 6466) were calculated based on data from the nationwide population-based Swedish Melanoma Register 2005–2013 with a follow-up through 2018. Results: Stages IIB and IIC showed significant differences in standardized cure proportions vs. stage IIA CM (0.80 (95% CI 0.77–0.83) stage IIA; 0.62 (95% CI 0.59–0.66) stage IIB; 0.42 (95% CI 0.37–0.46) for stage IIC). Significant differences in standardized cure proportions were found for stages IIIB and IIIC-D CM vs. stage IIIA (0.76 (95% CI 0.68–0.84) stage IIIA; 0.52 (95% CI 0.45–0.59) stage IIIB; 0.35 (95% CI 0.30–0.39) for stage IIIC–D). Conclusions: The results are emphasizing the poor prognosis with low proportions cured by surgery only for sub-groups of stage II–III CM, specifically within stages IIB–C CM.

## 1. Introduction

The disease-specific survival in cutaneous melanoma (CM) is highly dependent on early detection and surgical removal of the primary tumor as advanced disease predicts worse patient outcomes [1]. Overall, the 5-year relative survival is over 90% [2]. Specifically, the disease-specific survival rates range from 63–81% in stage II CM and from 36–63% in stage III CM.

Immune checkpoint inhibitors and targeted therapy have improved the outcome in the adjuvant setting of resected stage III CM [3,4]. For example, after 42 months of follow-up, the PD-1-inhibitor pembrolizumab vs. placebo has conveyed a 40% risk reduction of distant metastases in resected stage III disease [4]. Adjuvant nivolumab decreased the risk of recurrences by 29% as compared to adjuvant treatment with the CTLA-4-inhibitor ipilimumab in surgically resected stage III or IV CM after 28 months follow-up [5,6,7]. These therapies have also shown promising results in the neoadjuvant setting of stage III CM [8,9,10]. The efficacy of adjuvant therapies is yet to be established for resected stage II CM and results from ongoing adjuvant clinical trials may lead to changes in the therapeutic approach.

Stage II–III CM patients are at high risk of relapse with a heterogeneous outcome, but not all experience excess mortality due to their disease [1,11]. The cure proportion represents the proportion of patients who experience the same mortality rate as the general population and can be estimated using cure models [12,13]. Based on the American Joint Committee on Cancer (AJCC) 7th classification, we have previously shown that the cure proportion and survival among uncured for stage III CM patients were significantly higher in patients diagnosed with non-ulcerated stage III CM compared to ulcerated stage III CM and stage IV disease after adjusting for age, sex and tumor site [13]. Since then, the 8th version of the AJCC classification for CM has been introduced and specifically changed the stage III staging system based on the T-stage, as well as the ulceration status of the primary CM, and of the nodal status including satellite and/or in transit metastases (See detailed staging information specifically for stage II–III CM described by J.E. Gershenwald and R.A. Scoyler in the Annals of Surgical Oncology 2018) [11,14]. Isaksson et al. [15] found that a high proportion of stage III CM patients was restaged when reclassified according to the AJCC 8th edition. A recent European report has shown that the CM-specific survival rates for stage III CM based on the Central Malignant Melanoma Registry (CMMR) and European Organisation for Research and Treatment of Cancer (EORTC) were less favorable as compared to those based on the AJCC8 [16]. There is increasing evidence to use biomarkers for ICI response in CM e.g., tumors with high levels of tumor mutational burden can benefit from immunotherapy, but there is a need for future biomarker research [17,18].

There is thus an unmet need to characterize CM patients at high risk of recurrences also with regard to selection for adjuvant systemic therapy. In this study, we aimed to follow-up and estimate the cure proportion and survival among CM patients in AJCC8 stage II–III in Sweden by using data from the nationwide population-based Swedish Melanoma Registry (SweMR) between 2005 and 2013 with a follow-up through 2018.

## 2. Results

### 2.1. Clinicopathologic Characteristics

Patients and clinical characteristics are presented in Table 1. Between 2005 and 2013, a larger proportion of the patients were diagnosed with stage II CM (79.7%; *n* = 5156) compared to stage III (20.3%; *n* = 1310) in Sweden. Stage IIA was the most common substage (41.0%) within the stage II group, and stage IIIC the most common substage (56.0%) within the stage III group. The median age at diagnosis was higher among stage II CM patients (72 years) compared to stage III patients (64 years). The majority of stage II CM patients were >70 years (54.2%; *n* = 2796), whereas a larger proportion within the stage III group was aged 51–70 years at diagnosis (42.7%; *n* = 560). In both stage groups, there was a slightly higher proportion of males being diagnosed with stage II CM (53.8%; *n* = 2773) and stage III CM (59.8%; *n* = 783), respectively. The median Breslow thickness of the primary CM was 3.3 mm in stage II patients and 3.1 mm in stage III patients, respectively. Out of 5156 stage II patients, almost 50 percent of the patients presented with T3 tumors; T3a (27.1%; *n* = 1401) or T3b (22.5%; *n* = 1161). This corresponded to T4b CM (26.2%; *n* = 343) in the stage III group. Ulceration was present in 60.2% (*n* = 3106) of the primary CM in the stage II group, compared to 51.2 percent (*n* = 671) in stage III CM. NM (48.2%; *n* = 2486) was the most common histologic subtype of the primaries for stage II CM, but SSM (40.5%; *n* = 530) and NM (43.0%; *n* = 563) were almost equally distributed in the stage III group.

### 2.2. Relative Survival Ratios, Cure Proportions and Median Survival Times

**Figure 1:**
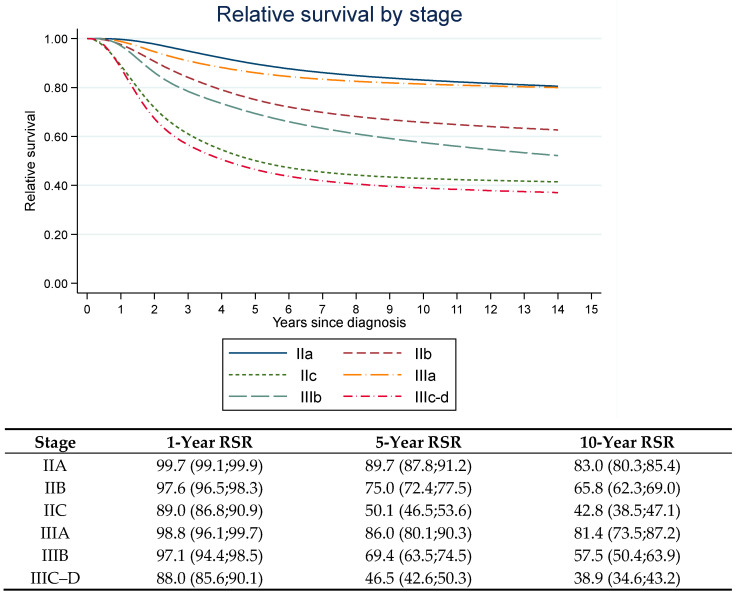
Cumulative relative survival by stage for patients diagnosed with stage II–III cutaneous melanoma in Sweden, 2005–2013.

Crude (i.e., unadjusted) relative survival ratio (RSR) for stage II–III are presented in Figure 1. The cumulative unadjusted survival was similar for stages IIA and IIIA, IIB and IIIB as well as for stages IIC and IIIC–D.

**Figure 2:**
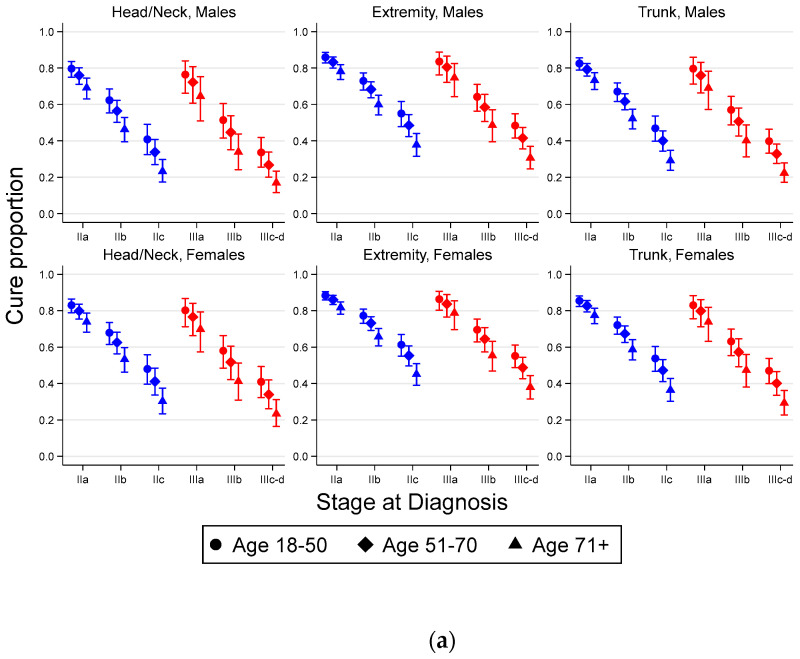

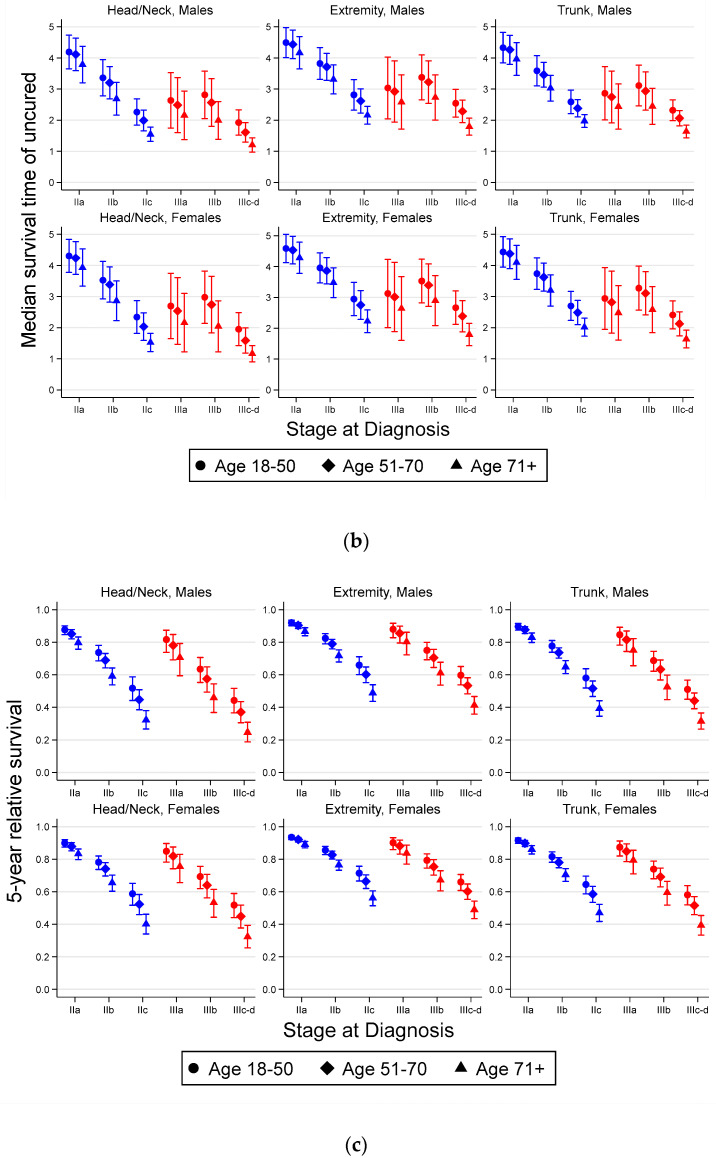
Estimated (**a**) cure proportions, (**b**) median survival time of uncured and (**c**) 5-year relative survival (with 95% confidence intervals), for men and women, respectively, diagnosed with stage II–III cutaneous melanoma in Sweden, 2005–2013.

Standardized (model based standardization over the age group, sex and tumor site distribution in the whole cohort) 1-year RSR, 5-year RSR, cure proportions, median survival time (MST) of uncured patients (measured in years) along with differences in the standardized measures (with 95% CI) by stage for stage II–III CM are presented in Table 2 (reference within each stage group) as well as in Table S1 (stage IIA as reference for all stage groups) and Table S2 (the corresponding stage II as reference for stage III). Estimates of the cure proportion, 1-year RSR, 5-year RSR and MST for uncured for each combination of age, sex, tumor site and stage are presented in Figure 2 and Figure S1.

Significant differences in standardized cure proportions were found within stages IIB and IIC, respectively, as compared to stage IIA; with a standardized cure proportion of 0.80 (95% CI 0.77–0.83) for stage IIA and 0.62 (95% CI 0.59–0.66) for stage IIB (difference (ref. stage IIA) 0.17; 95% CI 0.13–0.22) and 0.42 (95% CI 0.37–0.46) for stage IIC (difference (ref. stage IIA) 0.38; 95% CI 0.33–0.43) (Table 2). Significant differences in standardized cure proportions were also found for stages IIIB and IIIC–D CM in relation to stage IIIA; with a standardized cure proportion of 0.76 (95% CI 0.68–0.84) for stage IIIA, 0.52 (95% CI 0.45–0.59) for stage IIIB (difference (ref. stage IIIA) 0.25; 95% CI 0.14–0,35) and 0.35 (95% CI 0.30–0.39) for stage IIIC–D (difference (ref. stage IIIA) 0.42; 95% CI 0.33–0.51).

There was a significant difference in standardized MST of uncured patients within stage II CM group (Table 2), where standardized MST was approximately 0.8 months longer for stage IIA as compared to IIB and 2.0 months longer for stage IIA vs. IIC. There were no significant differences in standardized MST of uncured patients for IIIB CM compared to IIIA, or IIIC-D compared to IIIA. However, the standardized MST of uncured patients was similar between stages IIC and IIIA–B.

The standardized 5-year RSR were slightly higher than the standardized cure proportions for both stage II and stage III, respectively, and the differences in standardized 5-year RSR were smaller compared to the corresponding differences in cure proportions (Table 2). The difference (ref. IIA) for stage IIB in 5-year RSR was 0.14 (95% CI 0.11–0.17) and the difference (ref. IIA) for stage IIC in 5-year RSR was 0.35 (95% CI 0.31–0.39). For stage III CM, the differences (ref. stage IIIA) in 5-year RSR were 0.18 (95% CI 0.10–0.26) for stage IIIB and 0.36 (95% CI 0.29–0.43) for stage IIIC.

A statistically significant difference in the standardized cure proportion was shown for stages IIIB and IIIC–D vs. stage IIA (Table S1). A non-significant difference in standardized cure proportions was found for stage IIIA 0.76 (95% CI 0.68–0.84) vs. IIA 0.80 (95% CI 0.77–0.83) (difference (ref. stage IIA) 0.03, 95% CI −0.05–0.12). Despite this similarity, standardized MST of the uncured patients was significantly shorter for stage IIIA vs. IIA (difference 1.6; 95% CI 0.6–2.5) (Tables S1 and S2). There was a statistically significant difference in cure proportion between stages IIB vs. IIIB and IIC vs. IIIC/D (Table S2). Standardized MST of the uncured for stage IIIA was, in turn, comparable with that for stage IIIB. However, there was not a significant difference in the 5-year RSR between stages IIIA vs. IIA, but significantly lower 5-year RSR were found for stages IIB–C and IIIB–C/D compared to stage IIA and also for stages IIIB and IIIC/D as compared to the corresponding stage II group (Tables S1 and S2).

Based on the sensitivity analyses where we investigated potential interactions between stage and the other covariates, we found an interaction effect between stage and tumor site. The estimates of interest were, however, similar to the main model, except for the results for stage IIIA that were lower, especially for tumors on the head/neck CM. Given the small number of individuals in this group, we chose to use the model without interactions as the main model. We did not find a difference in standardized cure proportions between nodular melanoma (NM) and other histological types, and the standardized estimates for stage at diagnosis were similar to the main model when including histopathological subtype (NM vs. other).

## 3. Discussion

Previously published reports evaluating CM-specific survival based on the 8th AJCC version of CM staging have mainly used traditional methods for survival analyses. This is, to our knowledge, the first report showing cure proportions for stage II–III CM based on AJCC 8. We found that the cure proportions for all sub-groups of stage II–III CM were lower than the 5-year RSR, indicating that patients experience an excess mortality beyond 5 years from primary surgery. Importantly, low proportions were cured by surgery only for sub-groups of stage II–III CM, specifically within stage IIB–C CM. In the present study, the majority of the patients did not receive adjuvant systemic therapy (which was introduced as standard of care in Sweden for resected stage III CM from late 2018 and during 2019). The use of effective adjuvant systemic therapies emphasizes the interest in estimating the probability of cure where estimates of both the cure proportion and the survival distribution for those uncured show differences in survival between groups that cannot readily be detected from standard summary measures, for example 5-year RSR. The cure proportion is in this context a measure of patient’s benefit. In the 4-year follow-up of the Combi-AD trial, the effect from adjuvant targeted therapy in resected BRAFV600-mutant CM was approximately comparable to that of adjuvant anti-PD1 therapy [19]. Cure estimates have also been presented in the Combi-AD trial showing a cure rate of 54 percent (95% CI, 49–59%) in the dabrafenib plus trametinib arm vs. 37 percent (95% CI, 32–42%) in the placebo arm in the adjuvant setting of resected stage III CM.

Patients with resected stage II–III CM have an increased risk of recurrences and death. Approximately 14 percent of patients with a high-risk primary CM develop recurrences within two years which has been associated with features such as head-neck location of the primary CM and sentinel node biopsy positivity [20]. However, almost 50% of the patients were diagnosed with stage IB CM which could have reduced the actual proportion of recurrences. Swedish population-based data have demonstrated a five-year recurrence-free survival (RFS) of 66.2% for T3 CM and a RFS of 51.4% for T4 CM. In the multivariate analysis, recurrences were 6.7 times higher in T4 CM patients as compared to T1 CM patients with a median time of 0.8 years until recurrences occurred for stage IIB-C CM. In stage II disease, recurrences are frequently patient detected showing the importance of educating self-examinations [21]. Stage IIA or IIIA disease have a reported 5-year CM-specific survival of 94% or 93%, respectively [11]. Patients with stage IIC disease have a worse prognosis than those with stage IIIA disease. The 5-year CM-specific of stage IIC (82%) is comparable to stage IIIB (83%). However, the crude 5-year relative survival in our cohort was lower per stage group as compared the results from CM staging according to the 8th AJCC version. Our results (i.e., median CM thickness, median age of the CM patients as well as the RSR in our study as compared to the CM-specific survival) were comparable to the findings by Garbe et al. [16] based on the CMMR. Differences between studies could reflect different distribution of prognostic factors in previous reports, such as age, sex, and T-stage, between the populations. Moreover, a complete follow-up of CM-related deaths is of importance to evaluate and not overestimate the CM-survival and these are fully covered in the population-based SweMR. Interestingly, in the present study, stages IIA/IIIA, IIB/IIIB and IIC/IIIC–D followed the same pattern for the crude (unadjusted) RSR. Moreover, the adjusted analyses seem to emphasize the poor prognosis, specifically of stage IIB-C CM. However, ongoing clinical trials have yet to show if adjuvant therapy for stage II patients improves the outcome in this setting.

NM was the most common histopathologic subtype of CM for both stage II and stage III CM in the present study. This is in line with published data that most CM over 2 mm in tumor thickness are NM and also that the majority of NM are thicker than 2 mm at diagnosis [22]. NM growth pattern may have an impact on the risk of death in the disease but also in patients with metastatic disease receiving targeted therapy. However, we did not find a difference in cure proportions nor stage at diagnosis between NM and other histological subtypes of CM. Likewise, there are known differences in CM survival with a superior survival for women [23,24,25,26]. We could confirm these prognostic differences also in this cohort.

The strengths of this study include the population-based, nationwide patient cohort with high-coverage CM data and with complete and long-term follow-up of survival status. The cure models have potential limitations such as that the models will estimate a cure proportion even when statistical cure is not reached. This was the reason to only include patients diagnosed up until 2013, since follow-up information was available until 31 December 2018. This yielded a potential follow-up of between 5 and 14 years for all patients.

## 4. Materials and Methods

### 4.1. Patients and Methods

Patients ≥18 years diagnosed with stage II–III CM between 2005 and 2013 without a previous invasive CM were identified from the SweMR (*n* = 6466). Patients with missing information on tumor site (*n* = 32) were excluded from the statistical analyses. For patients with multiple primary CM at diagnosis, the most advanced tumor was included in the analyses. The median follow-up was 5.6 years (range 0.02 to 13.99 years). All patients were observed from the date of diagnosis until death, emigration, or end of follow-up on 31 December 2018, whichever occurred first.

Stage at diagnosis was categorized as stage IIA–C and stage IIIA–D according to the AJCC 8 staging [11]. (See detailed staging information specifically for stage II–III CM described by J.E. Gershenwald and R.A. Scoyler in the Annals of Surgical Oncology 2018 [14]). Information on age (18–50, 51–70, >70 years), sex, tumor site (upper extremity, lower extremity, acral sites, trunk, head-neck), tumor thickness according to Breslow, T-stage (T1-4a/b), ulceration status (present, absent, unknown information) and histologic type NM, superficial spreading melanoma (SSM), lentigo malignant melanoma (LMM), acral lentiginous melanoma (ALM), other, unknown information) was obtained from the SweMR.

### 4.2. Statistical Methods

The statistical analysis was performed in a similar way as in previous studies on cure for CM patients in Sweden [13,27,28]. A flexible parametric cure model within a relative survival setting was used to estimate 1- and 5-year RSR, cure proportions and the MST of uncured patients. In a relative survival setting, cause of death is not used; instead, the mortality due to melanoma is estimated as the excess mortality among the patients in comparison to the general population. In this study, the general population mortality was obtained from population life tables at the Human Mortality Database [29], and stratified by sex, age and calendar year. The model included the main effects of sex, age groups (18–50 years, 51–70, >70), tumor site (head & neck, trunk, extremities) and stage (IIA, IIB, IIC, IIIA, IIIB, IIIC–D), as well as allowing for time-varying effects for all the covariates. The splines used to model the underlying excess hazard had 7 knots, and 5 knots were used for the time-varying effects.

Based on the fitted model, standardized estimates of the 1-year/5-year RSR, cure proportion and MST of uncured patients were obtained for each of the stages. The standardized estimates were standardized to age group, sex and tumor site distribution in the whole cohort. The standardized estimates were interpreted as the average estimate within the whole cohort, given the age group, sex and tumor site, but if everyone had the same stage. The standardized estimates for each stage are therefore comparable in terms of age group, sex and tumor site and can be interpreted as adjusted estimates. To be able to draw conclusions about any significant differences in standardized estimates, we also present differences in standardized estimates together with 95% confidence intervals for the differences.

Flexible parametric cure models with interactions between stage and each of the other covariates were also fitted as sensitivity analyses. These models included the covariates and time-varying effects as described for the main model, as well as an interaction between stage and one of the other covariates (sex, age group, tumor site), giving three additional models (one for each covariate having an interaction with stage). Models including histological subtype categorized as NM vs. other were also fitted as sensitivity analyses, to see if the estimates for stage changed when additionally adjusting for histological subtype. These models also included age, sex, tumor site and stage as described above.

## 5. Conclusions

Our results clearly show the poor prognosis with low proportions cured by surgery only for sub-groups of stage II–III CM, specifically within stage IIB–C CM. Cure analyses may serve as a complement to more established survival analyses, e.g., distant-free survival, RFS and overall survival, as well as highlighting the patient benefit from adjuvant systemic therapy in clinical trials.

## Figures and Tables

**Table 1:** Clinicopathological characteristics of patients diagnosed with Stage II–III cutaneous melanoma in Sweden, 2015–2013.

Clinico-Pathological Characteristics	Stage II *n* (%)				Stage III *n* (%)				
	All	A	B	C	All	A	B	C	D
Number of patients	5156	2114 (41.0)	1810 (35.1)	1232 (23.9)	1310	210 (16.0)	321 (24.5)	733 (56.0)	46 (3.5)
Median age at diagnosis (years)	72				64				
Age groups (years)									
18–50	625 (12.1)	371 (59.4)	186 (29.8)	68 (10.9)	324 (24.7)	90 (27.8)	84 (25.9)	147 (45.4)	3 (0.9)
>50–70	1735 (33.7)	826 (47.6)	609 (35.1)	300 (17.3)	560 (42.7)	95 (17.0)	151 (27.0)	293 (52.3)	21 (3.8)
>70	2796 (54.2)	917 (32.8)	1015 (36.3)	864 (30.9)	426 (32.5)	25 (5.9)	86 (20.2)	293 (68.8)	22 (5.2)
Sex									
Men	2773 (53.8)	1101 (39.7)	1003 (36.2)	669 (24.1)	783 (60.0)	113 (14.4)	172 (22.0)	461 (58.9)	37 (4.7)
Women	2383 (46.2)	1013 (42.5)	807 (33.9)	563 (23.6)	527 (40.2)	97 (18.4)	149 (28.3)	272 (51.6)	9 (1.7)
Tumor site									
Upper/lower extremity, acral sites	2240 (43.4)	960 (42.9)	745 (33.3)	535 (23.9)	583 (44.5)	98 (16.8)	140 (24.0)	332 (56.9)	13 (2.2)
Trunk	1958 (38.0)	792 (40.4)	725 (37.0)	441 (22.5)	626 (47.8)	106 (16.9)	156 (24.9)	338 (54.0)	26 (4.2)
Head/neck	932 (18.1)	349 (37.4)	333 (35.7)	250 (26.8)	95 (7.3)	5 (5.3)	23 (24.2)	60 (63.2)	7 (7.4)
Unknown information	26 (0.5)	13 (50.0)	7 (26.9)	6 (23.1)	6 (0.5)	1 (16.7)	2 (33.3)	3 (50.0)	0 (0)
Median tumor thickness (mm)	3.3				3.1				
T-stage									
T1–T2a	-	-	-	-	302 (23.1)	210 (69.5)	56 (18.5)	36 (11.9)	-
T2b	713 (13.8)	713 (100)	-	-	77 (5.9)	-	65 (84.4)	12 (15.6)	-
T3a	1401 (27.1)	1401 (100)	-	-	217 (16.6)	-	200 (92.2)	17 (7.8)	-
T3b	1161 (22.5)	-	1161 (100)	-	238 (18.2)	-	-	238 (100)	-
T4a	649 (12.6)	-	649 (100)	-	133 (10.2)	-	-	133 (100)	-
T4b	1232 (23.9)	-	-	1232	343 (26.2)	-	-	297 (86.9)	46 (13.4)
Histologic subtype									
NM	2486 (48.2)	700 (28.2)	968 (38.9)	818 (32.9)	563 (43.0)	33 (5.9)	104 (18.5)	396 (70.3)	30 (5.3)
SSM	1714 (33.2)	977 (57)	526 (30.7)	211 (12.3)	530 (40.5)	140 (26.4)	155 (29.2)	224 (42.3)	11 (0.6)
LMM	237 (4.6)	111 (46.8)	75 (31.6)	51 (21.5)	27 (2.1)	7 (18.9)	9 (24.3)	11 (29.7)	0 (0)
ALM	96 (1.9)	33 (34.4)	37 (38.5)	26 (27.1)	31 (2.4)	1 (2.7)	9 (29.0)	21 (67.7)	0 (0)
Other	617 (12.0)	291 (47.2)	201 (32.6)	125 (20.3)	156 (11.9)	29 (18.9)	44 (28.2)	79 (50.6)	4 (2.6)
Unknown information		2 (33.3)	3 (50.0)	1 (16.7)	3 (0.2)	0 (0)	0 (0)	2 (66.7)	1 (33.3)

T-stage and stage according to American Joint Committee on Cancer (AJCC) 8th version for cutaneous melanoma (See detailed staging information specifically for stage II–III CM described by J.E. Gershenwald and R.A. Scoyler in the Annals of Surgical Oncology 2018 [14]). SSM: superficial spreading melanoma; NM: nodular melanoma; LMM: lentigo malignant melanoma; ALM: acral lentiginous melanoma.

**Table 2:** Standardized 1-year relative survival ratios (RSR), 5-year RSR, cure proportions and median survival times (MST) of uncured with 95% confidence intervals (CI) for patients diagnosed stage II–III * cutaneous melanoma in Sweden, 2005–2013.

Stage	Standardized 1-Year RSR (95% CI)	Difference in Standardized 1-Year RSR	Standardized 5-Year RSR (95% CI)	Difference in Standardized 5-Year RSR	Standardized Cure Proportion (95% CI)	Difference Standardized Cure Proportion	Standardized MST (Years) of Uncured (95% CI)	Difference in MST
IIA	1.00 (0.99;1.00)	-	0.88 (0.86;0.89)	-	0.80 (0.77;0.83)	-	4.2 (3.8;4.7)	-
IIB	0.97 (0.96;0.98)	0.03 (0.02;0.04)	0.74 (0.71;0.76)	0.14 (0.11;0.17)	0.62 (0.59;0.66)	0.17 (0.13;0.22)	3.4 (3.1;3.7)	0.8 (0.3;1.4)
IIC	0.90 (0.88;0.92)	0.09 (0.08;0.11)	0.52 (0.49;0.56)	0.35 (0.31;0.39)	0.42 (0.37;0.46)	0.38 (0.33;0.43)	2.3 (2.1;2.5)	2.0 (1.5;2.4)
IIIA	0.98 (0.96;1.00)	-	0.82 (0.75;0.88)	-	0.76 (0.68;0.84)	-	2.7 (1.8;3.5)	-
IIIB	0.95 (0.93;0.98)	0.03 (−0.006; 0.06)	0.64 (0.58;0.69)	0.18 (0.10;0.26)	0.52 (0.45;0.59)	0.25 (0.14;0,35)	2.8 (2.2;3.4)	−0.18 (−1.23;0.87)
IIIC–D	0.85 (0.82;0.88)	0.13 (0.10;0.16)	0.45 (0.41;0.49)	0.36 (0.29;0.43)	0.35 (0.30;0.39)	0.42 (0.33;0.51)	1.9 (1.7;2.1)	0.74 (−0.14;1.63)

* Reference: Stage IIA for the stage II cutaneous melanoma group and stage IIIA for the stage III cutaneous melanoma group.

## Data Availability

Data are available from the corresponding authors.

## Supplementary Materials

The following are available online at https://www.mdpi.com/article/10.3390/cancers13102456/s1, Table S1: Standardized 1-year relative survival ratios (RSR), 5-year RSR, cure proportions and median survival times (MST) of uncured with 95% confidence intervals (CI), for patients diagnosed with stage II–III * cutaneous melanoma in Sweden, 2005–2013, Table S2: Standardized 1-year relative survival ratios (RSR), 5-year RSR, cure proportions and median survival times (MST) of uncured with 95% confidence intervals (CI), for patients diagnosed with stage II–III * cutaneous melanoma in Sweden, 2005–2013, Figure S1: Estimates of the 1-year relative survival for each combination of age, sex, tumor site and stage.

## Abbreviation

Cutaneous Melanoma (CM); the American Joint Committee on Cancer (AJCC) classification; Central Malignant Melanoma Registry (CMMR) and European Organisation for Research and Treatment of Cancer (EORTC); Swedish Melanoma Register (SweMR); Relative Survival Ratio (RSR); Median Survival Tiume (MST); Nodular Melanoma (NM); Superficial Spreading Melanoma (SSM); Lentigo Malignant Melanoma (LMM); Acral Lentiginous Melanoma (ALM).
