# Supplementary material for: The Proportion Cured of Patients with Resected Stage II–III Cutaneous Melanoma in Sweden
Source: Cancers (Basel). 2021 May 18;13(10):2456. doi: 10.3390/cancers13102456 (PMC8158378; doi:10.3390/cancers13102456)

Supplementary material

Supplementary table 1. Standardized 1-year relative survival ratios (RSR), 5-year RSR, cure proportions and median survival times (MST) of uncured with 95% confidence intervals (CI), for patients diagnosed with stage II-III* cutaneous melanoma in Sweden, 2005-2013.

Stage	Standardized 1-year RSR (95% CI)	Difference in standardized 1-year RSR	Standardized 5-year RSR (95% CI)	Difference in standardized 5-year RSR	Standardized cure proportion (95% CI)	Difference standardized cure proportion	Standardized MST (years) of uncured (95% CI)	Difference in MST
**IIA**	1.00 (0.99;1.00)	-	0.88 (0.86;0.89)	-	0.80 (0.77;0.83)	-	4.2 (3.8;4.7)	-
**IIB**	0.97 (0.96;0.98)	0.03 (0.02;0.04)	0.74 (0.71;0.76)	0.14 (0.11;0.17)	0.62 (0.59;0.66)	0.17 (0.13;0.22)	3.4 (3.1;3.7)	0.8 (0.3;1.4)
**IIC**	0.90 (0.88;0.92)	0.09 (0.08;0.11)	0.52 (0.49;0.56)	0.35 (0.31;0.39)	0.42 (0.37;0.46)	0.38 (0.33;0.43)	2.3 (2.1;2.5)	2.0 (1.5;2.4)
**IIIA**	0.98 (0.96;1.00)	0.02 (-.007;0.04)	0.82 (0.75;0.88)	0.06 (-.003;0.12)	0.76 (0.68;0.84)	0.03 (-.05;0.12)	2.7 (1.8;3.5)	1.6 (0.6;2.5)
**IIIB**	0.95 (0.93;0.98)	0.04 (0.02;0.07)	0.64 (0.58;0.69)	0.24 (0.18;0.30)	0.52 (0.45;0.59)	0.28 (0.20;0.35)	2.8 (2.2;3.4)	1.4 (0.7;2.1)
**IIIC-D**	0.85 (0.82;0.88)	0.15 (0.12;0.17)	0.45 (0.41;0.49)	0.42 (0.38;0.47)	0.35 (0.30;0.39)	0.45 (0.40;0.50)	1.9 (1.7;2.1)	2.3 (1.8;2.8)

* Stage IIA cutaneous melanoma is reference for both stage II and III.

Supplementary table 2. Standardized 1-year relative survival ratios (RSR), 5-year RSR, cure proportions and median survival times (MST) of uncured with 95% confidence intervals (CI), for patients diagnosed with stage II-III* cutaneous melanoma in Sweden, 2005-2013.

**Stage**	**Standardized 1-year RSR (95% CI)**	**Difference in standardized 1-year RSR**	**Standardized 5-year RSR (95% CI)**	**Difference in standardized 5-year RSR**	**Standardized cure proportion (95% CI)**	**Difference standardized cure proportion**	**Standardized MST (years) of uncured (95% CI)**	**Difference in MST**
**IIA**	1.00 (0.99;1.00)	-	0.88 (0.86;0.89)	-	0.80 (0.77;0.83)	-	4.2 (3.8;4.7)	-
**IIB**	0.97 (0.96;0.98)	-	0.74 (0.71;0.76)	-	0.62 (0.59;0.66)	-	3.4 (3.1;3.7)	-
**IIC**	0.90 (0.88;0.92)	-	0.52 (0.49;0.56)	-	0.42 (0.37;0.46)	-	2.3 (2.1;2.5)	-
**IIIA**	0.98 (0.96;1.00)	0.02 (-.007;0.04)	0.82 (0.75;0.88)	0.06 (-.003;0.12)	0.76 (0.68;0.84)	0.03 (-.05;0.12)	2.7 (1.8;3.5)	1.6 (0.6;2.5)
**IIIB**	0.95 (0.93;0.98)	0.02 (-0.01;0.05)	0.64 (0.58;0.69)	0.10 (0.04;0.16)	0.52 (0.45;0.59)	0.11 (0.03;0.18)	2.8 (2.2;3.4)	0.56 (-0.13;1.2)
**IIIC-D**	0.85 (0.82;0.88)	0.05 (0.02;0.08)	0.45 (0.41;0.49)	0.07 (0.02;0.12)	0.35 (0.30;0.39)	0.07 (0.01;0.13)	1.9 (1.7;2.1)	0.35 (0.08;0.62)

*The corresponding stage II cutaneous melanoma s reference for stages IIIA-C/D.

Supplementary figure 1. Estimates of the 1-year relative survival for each combination of age, sex, tumor site and stage.

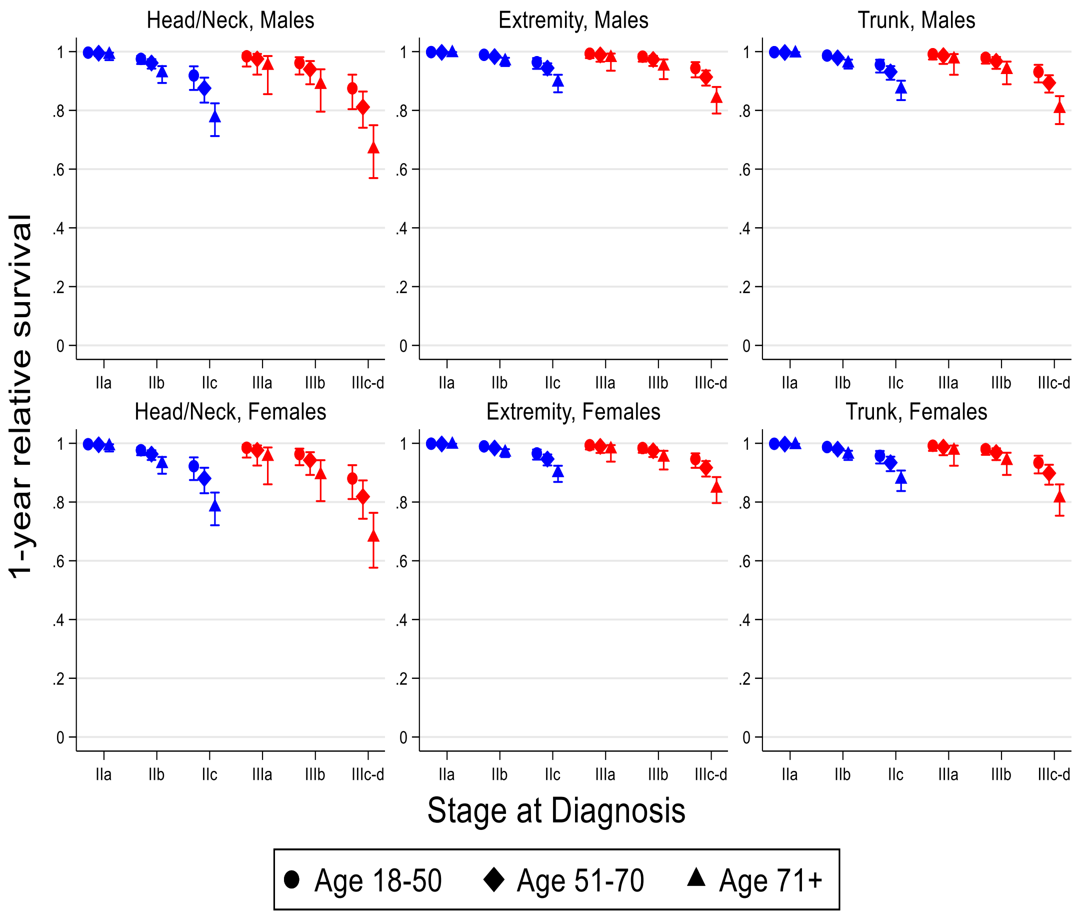

Supplement: Supplementary file 1 [file cancers-13-02456-s001.zip › cancers-1179792-supplementary.pdf]
